# Meeting Summary of The NYO3 5th NO-Age/AD Meeting and the 1st Norway–UK Joint Meeting on Aging and Dementia: Recent Progress on the Mechanisms and Interventional Strategies

**DOI:** 10.1093/gerona/glae029

**Published:** 2024-01-30

**Authors:** He-Ling Wang, Richard Siow, Tomas Schmauck-Medina, Jianying Zhang, Per Morten Sandset, Clare Filshie, Øystein Lund, Linda Partridge, Linda Hildegard Bergersen, Lene Juel Rasmussen, Konstantinos Palikaras, Ioannis Sotiropoulos, Jon Storm-Mathisen, David C Rubinsztein, Maria Grazia Spillantini, Chris I De Zeeuw, Leiv Otto Watne, Martin Vyhnalek, Katerina Veverova, Kristina Xiao Liang, Nektarios Tavernarakis, Vilhelm A Bohr, Koutaro Yokote, Janna Saarela, Hilde Nilsen, Efstathios S Gonos, Morten Scheibye-Knudsen, Guobing Chen, Hisaya Kato, Geir Selbæk, Tormod Fladby, Per Nilsson, Anne Simonsen, Dag Aarsland, Sofie Lautrup, Ole Petter Ottersen, Lynne S Cox, Evandro F Fang

**Affiliations:** Department of Clinical Molecular Biology, University of Oslo and Akershus University Hospital, Lørenskog, Norway; School of Cardiovascular and Metabolic Medicine & Sciences, Faculty of Life Sciences & Medicine, King's College London, London, UK; Department of Clinical Molecular Biology, University of Oslo and Akershus University Hospital, Lørenskog, Norway; Department of Clinical Molecular Biology, University of Oslo and Akershus University Hospital, Lørenskog, Norway; Xiangya School of Stomatology, Central South University, Changsha, Hunan, China; Department of Haematology, Oslo University Hospital, Oslo, Norway; Institute of Clinical Medicine, Faculty of Medicine, University of Oslo, Oslo, Norway; British Embassy Oslo, Oslo, Norway; Royal Norwegian Embassy in London, London, UK; Max Planck Institute for Biology of Ageing, Cologne, Germany; Department of Genetics, Evolution and Environment, Institute of Healthy Ageing, University College London (UCL), London, UK; Brain and Muscle Energy Group, Institute of Oral Biology, University of Oslo, Oslo, Norway; Center for Healthy Aging, Department of Neuroscience and Pharmacology, Faculty of Health Sciences, University of Copenhagen, Copenhagen, Denmark; Department of Cellular and Molecular Medicine, Center for Healthy Aging, University of Copenhagen, Copenhagen, Denmark; Department of Physiology, Medical School, National and Kapodistrian University of Athens, Athens, Greece; Institute of Biosciences and Applications NCSR “Demokritos,” Athens, Greece; Life and Health Sciences Research Institute (ICVS), School of Medicine, University of Minho, Campus de Gualtar, Braga, Portugal; Division of Anatomy, Department of Molecular Medicine, Institute of Basic Medical Sciences, University of Oslo, Oslo, Norway; Department of Medical Genetics, Cambridge Institute for Medical Research, University of Cambridge, Cambridge, UK; UK Dementia Research Institute, University of Cambridge, Cambridge, UK; Department of Clinical Neuroscience, University of Cambridge, Cambridge, UK; Netherlands Institute for Neuroscience, Amsterdam, The Netherlands; Department of Neuroscience, Erasmus MC, Rotterdam, The Netherlands; Institute of Clinical Medicine, Campus Ahus, University of Oslo, Oslo, Norway; International Clinical Research Centre, St. Anne’s University Hospital, Brno, Czech Republic; Department of Neurology, Second Faculty of Medicine, Charles University and Motol University Hospital, Prague, Czech Republic; Department of Neurology, Second Faculty of Medicine, Charles University and Motol University Hospital, Prague, Czech Republic; Department of Clinical Medicine, University of Bergen, Bergen, Norway; Institute of Molecular Biology and Biotechnology Foundation for Research and Technology, Heraklion, Greece; Medical School, University of Crete, Heraklion, Greece; Department of Cellular and Molecular Medicine, Center for Healthy Aging, University of Copenhagen, Copenhagen, Denmark; Laboratory of Molecular Gerontology, National Institute on Aging, National Institutes of Health, Baltimore, Maryland, USA; Department of Endocrinology, Hematology, and Gerontology, Chiba University Graduate School of Medicine, Chiba, Japan; Centre for Molecular Medicine Norway (NCMM), University of Oslo, Oslo, Norway; Institute for Molecular Medicine Finland (FIMM), HiLIFE, University of Helsinki, Helsinki, Finland; Department of Microbiology, Oslo University Hospital, Oslo, Norway; The Norwegian Centre on Healthy Ageing (NO-Age), Oslo, Norway; National Helenic Research Foundation, Institute of Biology, Medicinal Chemistry and Biotechnology, Athens, Greece; Department of Cellular and Molecular Medicine, Center for Healthy Aging, University of Copenhagen, Copenhagen, Denmark; Tracked.bio, Copenhagen, Denmark; Guangdong-Hong Kong-Macau Great Bay Area Geroscience Joint Laboratory, Guangzhou, China; Department of Microbiology and Immunology, School of Medicine; Institute of Geriatric Immunology, School of Medicine, Jinan University, Guangzhou, China; Department of Endocrinology, Hematology, and Gerontology, Chiba University Graduate School of Medicine, Chiba, Japan; Institute of Clinical Medicine, Faculty of Medicine, University of Oslo, Oslo, Norway; Norwegian National Centre for Ageing and Health, Vestfold Hospital Trust, Tønsberg, Norway; Institute of Clinical Medicine, Campus Ahus, University of Oslo, Oslo, Norway; Department of Neurology, Akershus University Hospital, Lørenskog, Norway; Department of Neurobiology, Care Sciences and Society, Division of Neurogeriatrics, Center for Alzheimer Research, Karolinska Institutet, Stockholm, Sweden; Department of Molecular Medicine, Institute of Basic Medical Sciences, University of Oslo, Oslo, Norway; Department of Molecular Cell Biology, Institute for Cancer Research, Oslo University Hospital Montebello, Oslo, Norway; Centre for Age-Related Medicine, Stavanger University Hospital, Stavanger, Norway; Department of Old Age Psychiatry, Institute of Psychiatry, Psychology, and Neuroscience, King’s College London, London, UK; Department of Clinical Molecular Biology, University of Oslo and Akershus University Hospital, Lørenskog, Norway; Centre for Sustainable Healthcare Education, Faculty of Medicine, University of Oslo, Oslo, Norway; Karolinska Institutet, Stockholm, Sweden; Department of Biochemistry, University of Oxford, Oxford, UK; Department of Clinical Molecular Biology, University of Oslo and Akershus University Hospital, Lørenskog, Norway; The Norwegian Centre on Healthy Ageing (NO-Age), Oslo, Norway; (Biological Sciences Section)

**Keywords:** Aging, Alzheimer’s disease, Dementia, Longevity, Neurodegeneration

## Abstract

Unhealthy aging poses a global challenge with profound healthcare and socioeconomic implications. Slowing down the aging process offers a promising approach to reduce the burden of a number of age-related diseases, such as dementia, and promoting healthy longevity in the old population. In response to the challenge of the aging population and with a view to the future, Norway and the United Kingdom are fostering collaborations, supported by a “Money Follows Cooperation agreement” between the 2 nations. The inaugural Norway–UK joint meeting on aging and dementia gathered leading experts on aging and dementia from the 2 nations to share their latest discoveries in related fields. Since aging is an international challenge, and to foster collaborations, we also invited leading scholars from 11 additional countries to join this event. This report provides a summary of the conference, highlighting recent progress on molecular aging mechanisms, genetic risk factors, DNA damage and repair, mitophagy, autophagy, as well as progress on a series of clinical trials (eg, using NAD^+^ precursors). The meeting facilitated dialogue among policymakers, administrative leaders, researchers, and clinical experts, aiming to promote international research collaborations and to translate findings into clinical applications and interventions to advance healthy aging.

Aging is emerging as a significant health challenge worldwide, including in Norway and the United Kingdom. The increase in the number of people reaching old age brings formidable healthcare and socioeconomic pressures globally (1–4). Dementia, especially Alzheimer’s disease (AD) which constitutes around 70% of dementia, is one of the most common diseases associated with aging, and imposes substantial pressure on patients, their families, and society as a whole (5). The geroscience hypothesis posits that slowing down the aging process may reduce the risk of developing age-related diseases, including dementia. In response to the aging challenge, collaborative work among stakeholders and countries is urgently needed. Both Norway and the United Kingdom are in the forefront of aging and dementia research, and there is a great opportunity to boost bilateral collaborations. Correspondingly, UK Research and Innovation (UKRI) and Research Council of Norway (RCN) have signed a Money Follows Cooperation agreement to reduce barriers to cross-border collaboration. Additionally, we call for collaborative research networks in aging biology and clinical translation via interdisciplinary and multiscale approaches following a new model established by UKRI in the United Kingdom (6).

On September 18–19, 2023, the 1st Norway–UK joint meeting on aging and dementia was held in Oslo, hosted by E.F.F. (University of Oslo and Akershus University Hospital, Norway), L.S.C. (University of Oxford, UK), and R.S. (King’s College London, UK). The meeting was opened by educational and political leaders of the 2 nations, including Prof. P.M.S. (Vice-Rector for Research and Innovation of the University of Oslo), C.F. (Deputy Head of Mission from the British Embassy in Oslo), and Ø.L. (Counselor for Research and Education from the Royal Norwegian Embassy in London). The meeting attracted over 40 experts and scholars from 13 countries (Figure 1), including China, Czech Republic, Denmark, Finland, Germany, Greece, Japan, The Netherlands, Norway, Portugal, Sweden, the United Kingdom, and the United States. They gathered to share the latest research findings on various aspects of global aging and to engage in discussions and workshops on topics including mechanisms of human aging (such as genetic risk factors, DNA damage and repair, mitophagy and autophagy, etc.), connections between aging processes and disease, and promoting a healthy aging environment. Importantly, discussions included potential interventions to reduce biological aging as well as the need for translation of research findings into clinical application. In this report, we group and summarize the presentations by overall theme.

**Figure 1.**
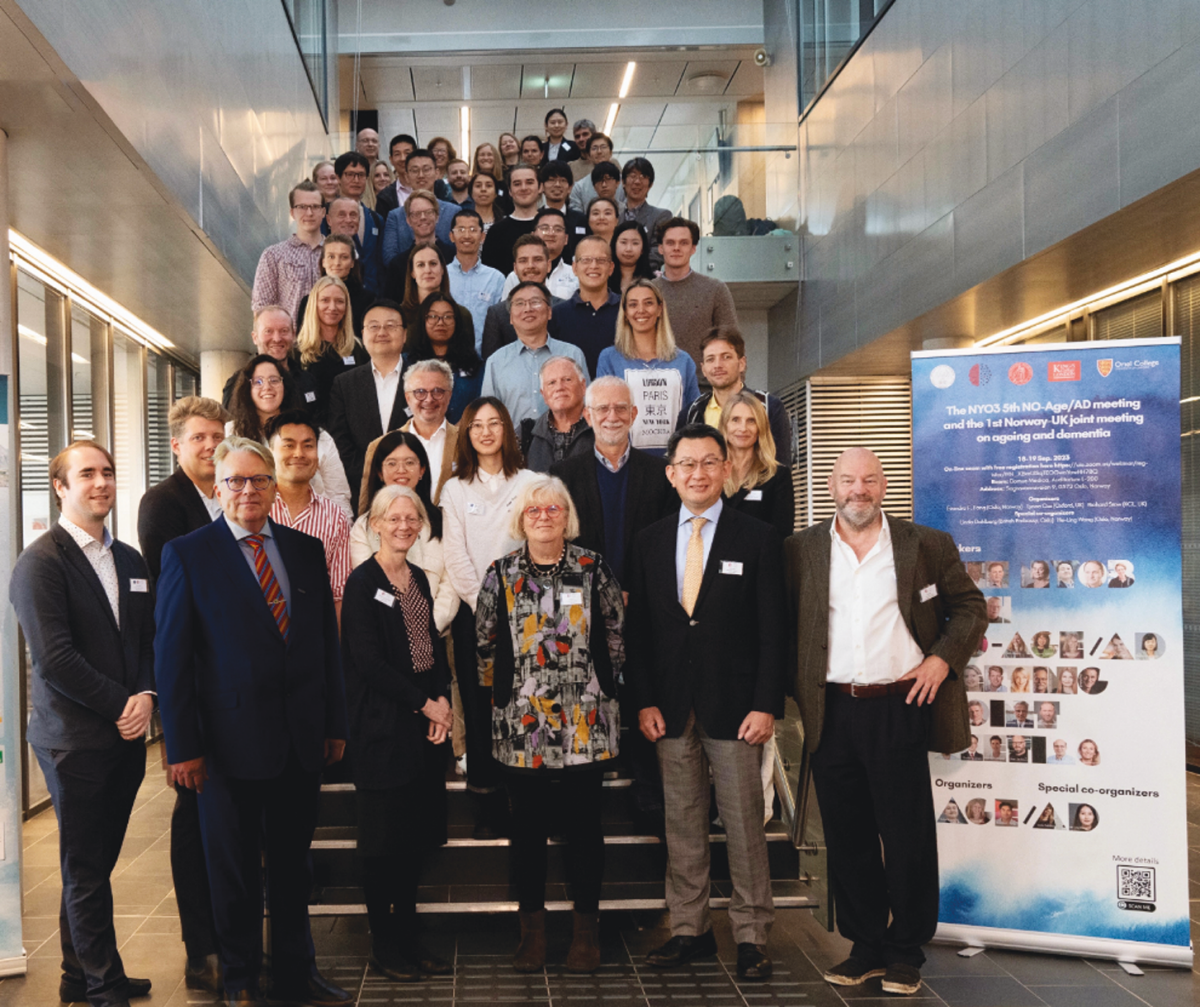
Group photo of The NYO3 5th NO-Age/AD meeting and the 1st Norway–UK joint meeting on aging and dementia.

## Hallmarks of Aging: Mechanisms and Linkages to Aging and Diseases

### mTOR and Aging

Following an overview of aging hallmarks and how they connect to age-related phenotypes and diseases, Prof. Dame Linda Partridge (University College London, UK) presented data on sexually dimorphic effects of rapamycin on nutrient-sensing and autophagy in both Drosophila and mice. Although age-related GI tract deterioration is found to be greater in female than male flies (7), female gut pathology can be reduced to male levels on rapamycin administration through stimulation of autophagy in gut enterocytes (8). Interestingly, male flies have higher levels of autophagy in the gut than females, potentially explaining the lack of effect of rapamycin on the aging male gut. Interestingly, her lab has demonstrated that brief rapamycin treatment early in the life course of flies is protective against both development of tumors and gut leakage later in life (9).

### Senescence

Lynne S. Cox, University of Oxford, UK, discussed the importance of senescent cells in aging and age-related diseases, summarizing a range of findings from the field, and highlighting the need for both senolytic and senomorphic approaches when developing therapies. The role of senescence in both chronic and infectious disease risk was briefly addressed, particularly in the context of the coronavirus disease (COVID) pandemic and high morbidity/mortality risk for those living with obesity, diabetes, or socioeconomic deprivation. Such individuals are likely to have elevated levels of senescent cells, which increases the risk both of severe acute respiratory coronavirus 2 (SARS-CoV-2) infection (as the senescence-associated secretory phenotype [SASP] enhances viral uptake) and of cytokine storm (10). She described findings from the Mayo clinic that senolytic treatments significantly reduced mortality in old mice infected with coronavirus (11,12) and that mTOR inhibitors, which act as senomorphics, support the older population's immune system (13–15) and show promise in reducing harm in nursing home residents exposed to SARS-CoV-2 (ClinicalTrials.gov ID NCT04409327). She discussed her lab’s current work on screening for agents that suppress the SASP, including optimization of a biosensor platform for SASP factor IL-6 (16). Finally, she discussed human clinical trials of senolytics in idiopathic pulmonary fibrosis, diabetic kidney disease, arthritis, and AD, as well as her collaborative work on a UK clinical trial of rapamycin assessing impacts on aging muscle and immune function.

### Genetic Risk Factors and DNA Damage

Lene Juel Rasmussen, University of Copenhagen, Denmark, raised the important question of why some people reach 100 years of lifespan in relatively good health, and the role of specific genes associated with high and low longevity including *Forkhead box O3* (*FOXO3)* and *apolipoprotein E* (*APOE)*. Her talk then focused on a novel FOXO3 target *Oxidative Stress Responsive Serine Rich 1* (*OSER1)*, a gene implicated in the oxidative stress response. *OSER1*, she found, extends lifespan in nematodes, silkworms, and fruit flies (unpublished data). They found that this gene is highly expressed in long-lived *Caenorhabditis elegans* in relation to the short-lived ones. On the other hand, knocking down *OSER1* shortens lifespan and affects the mitochondria. Phenotypically, *OSER1* SNPs were associated with long-lived humans.

Janna Saarela, University of Oslo, Norway, further explored extreme longevity, discussing the role of genetic, environmental, and lifestyle factors and highlighting specific variants associated with long life. Her discussion introduced the rare inherited premature aging syndromes Hutchinson–Gilford Progeria Syndrome (HGPS), and Werner Syndrome (WS), with underlying molecular defects related to laminopathy (HGPS) as well as DNA repair deficiency, respectively (17). Finally, she introduced the FinnGen study (18), which leverages Finland’s genetically isolated population for genetic insights, emphasizing the significance of Finland’s genetic history, the prevalence of loss-of-function variants, and key findings related to aging-related genes, such as FOXO3, particularly their associations with diseases and phenotypes, with an emphasis on cancer and other health conditions.

Vilhelm Bohr, University of Copenhagen, Denmark, discussed the critical role of DNA repair in healthy aging and brain health, emphasizing the importance of Poly [ADP-ribose] polymerase 1 (PARP1), which requires NAD^+^ as a cofactor for DNA repair (19). Nicotinamide adenine dinucleotide (oxidized form, NAD^+^) is a fundamental biological molecule, involved in energy production, metabolism, redox regulation, various cell signaling pathways, as well as in regulating cell survival and death (20). The molecular mechanisms underpinning the role of NAD^+^ in enhancing DNA repair were discussed, as well as the mitochondrial function of PARP1, and the use of premature aging models to study aging processes (19). He also presented data showing the efficacy of pharmacological upregulation of mitophagy (NAD^+^ supplementation (21) and urolithin A/UA,) in inhibiting AD pathologies of rodent models. He discussed ongoing research comparing the pathways involved in stimulation of mitophagy with nicotinamide riboside (NR) and urolithin A, both natural substances that are used clinically in various conditions and that have potential against AD.

Hilde Nilsen, Oslo University Hospital and University of Oslo, Norway, further explored the role of DNA damage in progressive neurodegenerative diseases and possible clinical interventions (22), using models of DNA repair deficiency syndromes to better understand aging mechanisms and common health conditions (such as frailty and neurodegeneration). Following the previous findings that hyper PARylation-induced depletion of NAD^+^ is associated with accelerated aging in *ATM*-deficient *C elegans* and mouse models (23), her lab has conducted a 2-year clinical study on the effect of supplementation of an NAD^+^ precursor NR in ataxia-telangiectasia (A-T) patients, a rare genetic disorder with premature aging features. She reported that NR improves neuro-motor function in A-T patients and may also delay disease progression when compared to historical control groups (24). These findings offer promise for potential clinical interventions for neurodegenerative diseases driven by DNA damage response and genomic instability.

### Autophagy and Mitophagy

Damaged or redundant macromolecules and organelles are recycled in healthy cells through autophagy, a stepwise pathway of recognition, engulfment, lysosomal degradation, and finally degradation, releasing building blocks for cellular reuse (25,26). Based on the mechanisms of cargo recognition and engulfment, autophagy is classified into macroautophagy (further divided into mitochondrial autophagy/mitophagy, ER-phagy, nucleophagy, etc.), chaperon-mediated autophagy, and microautophagy (25,27). Autophagy levels are generally reduced during aging and compromised autophagy is now recognized as a distinct hallmark of aging (previously included under “loss of proteostasis”) (25,28,29). Autophagy is thought to play a pivotal role in maintaining brain health, and hence the role of age-related dysfunctional autophagy in neurodegeneration was explored in this session of the meeting.

Konstantinos Palikaras, National and Kapodistrian University of Athens, Greece, discussed the impact of aging on mitochondrial homeostasis. Postmitotic neurons heavily rely on robust mitochondrial quality control to maintain their energy homeostasis and survival. Mitophagy, a form of selective mitochondrial autophagy, plays a pivotal role in neuronal development and survival during stress and aging (30,31). The deregulation of mitophagy and excessive mitochondrial damage are hallmark features of aging and neurodegenerative diseases (29,32,33). Neuronal mitophagy is a challenging event because the majority of mitochondria reside in distal neuronal processes, far away from the cell body, where mature lysosomes are primarily located. Despite spatial limitations, rapid mitochondrial elimination is essential for protecting neurons against cell death, highlighting the presence of localized quality control mechanisms to cope with mitochondrial stress and uphold neuronal function (34,35). While providing neuroprotection, precise regulation of mitophagy is crucial for cellular viability, as uncontrolled mitophagy can decrease mitochondrial numbers, cause energy depletion, and eventually result in cell demise (36,37). Understanding the molecular mechanisms governing neuronal mitophagy is pivotal, paving the way for developing novel mitophagy modulators that could affect cellular and organismal physiology in both health and disease.

Anne Simonsen, University of Oslo, Norway, reported that the mitochondrial matrix proteins 4-NIPSNAP1 (nitrophenylphosphatase domain and nonneuronal SNAP25-like protein homolog 1) and NIPSNAP2 accumulate on the mitochondria surface upon mitochondrial depolarization (38). There, they recruit proteins involved in selective autophagy, including autophagy receptors and ATG8 proteins, thereby functioning as an “opsonization” signal for mitophagy. NIPSNAP1 and NIPSNAP2 are predominantly expressed in different tissues and were shown to have a redundant function in mitophagy. Zebrafish lacking functional Nipsnap1 were reported to display reduced mitophagy in the brain and parkinsonian phenotypes, including loss of tyrosine hydroxylase (Th1)-positive dopaminergic neurons, reduced motor activity, and increased oxidative stress (38).

Sofie Lautrup, University of Oslo, Norway, presented work conducted in the E.F.F. group on the cellular-protective effects of NAD^+^ in the context of aging and AD (19,23,39). She highlighted the critical role of the NAD^+^-mitophagy axis in neuronal resilience, and its compromise in AD, noting that mitophagy is a viable target for drug intervention. The group also explored the potential of AI in screening drug candidates for AD (40). They are investigating the temporal-spatial changes in mitophagy in the brain during the aging process and in AD.

Further investigating the role of autophagy in neurodegeneration, D.C.R., University of Cambridge, UK, presented superresolution microscopy data visualizing the intricate process of autophagosome formation, as well as describing its significance in inhibiting neurodegeneration. Rubinsztein’s research revealed that autophagic precursor membranes emerge as finger-like structures from the RAB11A-recycling endosome compartment (41). Furthermore, he discussed how autophagy compromise contributes to neurodegenerative diseases, highlighting the potential of harnessing autophagy as a therapeutic strategy. He emphasized that autophagy plays a key role in the removal of toxic, aggregate-prone proteins, and that the upregulation of autophagy protects against cell stress and death in various neurodegenerative disease models. He outlined the role of microglia in mediating noncell-autonomous inhibition of neuronal autophagy in neurodegenerative diseases, demonstrating the complex interplay between different cell types in these conditions (42).

Nektarios Tavernarakis, University of Crete, Greece, addressed the mechanisms underpinning germline immortality versus somatic mortality. He discussed the accumulation of mutations in mitochondria in the soma during aging and how increased mitophagy in the soma promotes longevity (30). He further explored distortion of nuclear architecture and morphology with age, cancer, and progeroid HGPS, and the important role of nucleophagy in maintaining the integrity of the nucleus and the cell (43). Notably, he showed that knockdown of the autophagy protein Atg5 resulted in alterations in lamin B distribution and nuclear morphology, and that the autophagy protein Atg39 is present on nuclear envelope blebs that undergo nucleophagy. He discussed the role of nesprin-mediated autophagy/nucleophagy in mediating nuclear size and integrity; nesprins connect the nucleoskeleton to the cytoskeleton, colocalize with nucleolar fibrillarin, and interact with lipidated autophagy factor LC3-II. He showed that blocking the autophagy kinase ULK-1 resulted in altered nesprin localization, while depletion of nesprins led to the formation of micronuclei, giant nuclei, and nuclear protrusions. Interestingly, in the germline, nucleophagy was found to regulate nucleolar size (it is therefore of note that increased nucleolar size and prominence is a marker of senescent cells). In a worm model defective for nucleophagy (*anc-1* mutant), germline sterility was observed within only 5 generations, whereas mice homozygous mutant for nesprin 2 developed ovarian tumors; notably nesprin polymorphisms are associated with female infertility in humans. These data together strongly suggest that nucleophagy is required for germline immortality.

### Immune Aging

Guobing Chen, Jinan University, China, presented studies on both immune aging and autophagy. By using single-cell RNA and TCR sequencing of human blood samples across the lifespan, they show an age-dependent accumulation of transcriptome heterogeneity and variability in immune cells (44). Additionally, he explored the impact of aging on SARS-CoV-2 vaccine efficacy, noting delayed antibody production and weaker T-cell responses in older adults (45). The findings underscore the importance of understanding aging-related immune dynamics for optimizing vaccinations in the older population.

### Exposome

Richard Siow, King’s College London, UK, highlighted the importance of the exposome in human aging. This concept describes “the cumulative measure of environmental influences and associated biological responses throughout the lifespan . . .” (46), taking into account how factors such as lifestyles, social structures, built and natural environments, as well as temperature, interact with man’s biology to affect aging health. He further discussed the concept of nutrigenomics, that is, the possibility of optimizing a personalized diet based on an individual’s genetics.

## Age-Related Neurodegeneration

### Common Brain Diseases: Dementia and Delirium

Geir Selbæk, Norwegian National Centre for Ageing and Health, Vestfold Hospital Trust and University of Oslo, Norway, presented 2 significant studies in the field of cognitive impairment and dementia, both using data from the HUNT Study in Norway. The first study revealed higher-than-expected prevalence rates of dementia and mild cognitive impairment in Norway, emphasizing the need for preparation in healthcare and society as the numbers are projected to increase (47). The second study highlighted the association between midlife high blood pressure and later-life lower blood pressure in individuals with dementia, underscoring the importance of monitoring and managing blood pressure in older adults to mitigate dementia risk (48).

Per Nilsson, Karolinska Institutet, Stockholm, Sweden, works on molecular mechanisms of Aβ pathology in AD with a focus on impaired autophagy. Previously, he and his colleagues generated an amyloid precursor protein (App) knock-in mouse model that shows AD-associated Aβ pathology, serving as a good model for mechanistic studies on Aβ accumulation and their temporal appearance. Using this mouse model, his lab demonstrated that mitochondrial hypermetabolism precedes impaired autophagy and synaptic disorganization (49). More specifically, the identification of an accumulation of autophagosomes in the very synapses indicates that the synapse alterations and subsequent decreased performance of the synapses and impaired memory of the AD mice coincide with impaired autophagy in the synapses. This is in line with accumulated damaged mitochondria, including in the synapses, and compromised mitophagy in AD (21), again pointing to mitochondria as promising therapeutic targets.

Leiv Otto Watne from the Akershus University Hospital and the University of Oslo, Norway, explored the relationship between delirium and dementia, highlighting dementia as a high-risk factor for delirium. The study investigated the kynurenine pathway (KP), which links acute illness to cognitive issues through inflammation (50). Examining 586 hospitalized patients (248 with delirium), the study revealed elevated neurotoxic quinolinic acid in cerebrospinal fluid (CSF) during delirium, alongside increased KP metabolites. Quinolinic acid was associated with neuronal damage and strongly predicted 1-year mortality (positive correlation). The results indicate a robust connection between inflammation, neurotoxicity, and delirium through the kynurenine pathway, providing valuable insights for promising clinical application.

Chris I. De Zeeuw, Erasmus University Rotterdam, the Netherlands, discussed changes in the cerebellum, the orchestrator of behavior and other activities, in the development of AD. Mutations in the gene encoding presenilin-1 (PS1) are found in the vast majority of cases of familial Alzheimer’s disease (FAD), with some of these patients presenting cerebellar damage with amyloid plaques and ataxia with unclear pathophysiology (51). Carriers of the PS1-E280A allele show cerebellar motor deficits, which occur before dementia and even before occurrence of amyloid beta plaques. Such findings could be replicated in an APP/PS1 mouse model. Molecular analysis indicates impaired calcium homeostasis and mitochondrial dysfunction in PS1-E280A carriers, affecting neuronal activity and contributing to motor coordination deficits, which precede amyloid deposition and dementia. The study proposes that PS1-E280A influences both calcium homeostasis and amyloid precursor processing, contributing to FAD and neurodegeneration (51).

In addition to the common brain disorder AD, mechanisms and interventional studies of premature aging were also included. Koutaro Yokote and Hisaya Kato, Chiba University, Japan, discussed their ongoing research on WS, a rare genetic disorder characterized by premature aging. They detailed their approach to better understand the mechanisms of premature aging in WS and to develop potential therapeutic strategies (41). They reported success in establishing a WS case registry in Japan, revising diagnostic criteria, creating treatment guidelines, and contributing to WS’s designation as a national intractable disease. Using multiomics approaches, they analyzed patient samples and generated induced pluripotent stem cells (iPSCs) from WS patients, revealing the role of mitochondrial dysfunction in WS, consistent with their previous collaborative work with the Bohr and Fang labs (52), including the accumulation of lysine metabolites, a potential mitochondrial toxin. They also explored the potential of exon skipping using antisense oligonucleotides as a treatment strategy to restore the production of functional WRN protein. This approach will be of particular value in patients with the most common mutation in the WRN gene; they described promising outcomes in early-stage clinical studies.

### Tau and Tauopathies

Maria Grazia Spillantini, University of Cambridge, UK, discussed the importance of tau phosphorylation and neurofibrillary tangles in different types of neurodegenerations. She presented data indicating that astrocytes in P301S tau mice had lost their neuro-supportive ability, which could be rescued by thrombospondin in both the mouse model and in human iPSC-derived astrocytes. In addition, she showed that neurons containing tau aggregates expose phosphatidylserine on the outer leaflet of the plasma membrane (usually a sign of cells undergoing apoptosis), leading to their phagocytosis by microglia (53,54), but that it is possible to block this neuronal death by inhibiting an opsonin that aids phagocytosis. This could have twofold therapeutic benefits, preventing both neuronal loss and avoiding release of digested tau by microglia that would otherwise serve as nucleation centers for toxic tau aggregation. Her lab is also studying the importance of TMEM106B filaments (55) accumulation of which is a risk factor for frontotemporal dementia. Interestingly, TMEM106B is a lysosomal receptor mediating ACE-2-independent SARS-CoV-2 entry (56). Cleavage by a lysosomal protease releases an intracellular domain of TMEM106B, which leads to the formation of insoluble aggregates detectable from age 45 onwards. Importantly while TMEM106B is a ubiquitous protein, aggregates are only found in the brain. However, she reported that TMEM106B filaments, which can be labeled experimentally using oligothiophenes, do not colocalize with tau aggregates. They also found that TMEM106B aggregates accumulate in a strongly age-dependent but neurodegeneration-independent manner, suggesting that they may prove useful as aging.

Ioannis Sotiropoulos, Institute of Biosciences and Applications NCSR “Demokritos,” Greece, discussed a precision medicine-based holistic approach to AD etiopathogenesis and progression where different precipitating factors such as lifetime stress, chronic illness, inflammation, and chronic pain (57,58) can accelerate age-associated decreases in cognitive function and mood leading to AD. He focused on how chronic stress connects to brain aging and Tau pathology. Specifically, he presented that chronic stress and the main stress hormones, glucocorticoids, can dysregulate the endolysosomal and autophagic degradation mechanisms triggering Tau accumulation and related neuropathology and neuronal malfunction (59), highlighting the potential role of small extracellular vesicles and exosomes. He also emphasized the long presymptomatic time window (even 20 years long) between initiation of AD brain pathology and clinical symptoms/diagnosis describing the need for better brain biomarkers to overcome the “one size fits all” therapeutic approach and to proceed from stratified medicine to truly personalized medicine in AD. To this end, he presented new method of isolation of spontaneously released brain exosomes (60) and the promising use of neuronal-derived exosomes and Tau in peripheral blood (isolated through an neuronal marker-based pull-down protocol) in both AD diagnosis and prognosis of cognitive decline.

## Diagnosis and Interventions

### Biomarkers of Aging and Dementia

Morten Scheibye-Knudsen, University of Copenhagen, Denmark, addressed the limited understanding of age-related tissue changes in humans (61). Utilizing a vast data set of 33 million histological samples, he and colleagues have extracted numerous age- and mortality-related features from textual descriptions, referred to as The Human Pathome (pathoage.com). Their research revealed when pathological aging begins at both the organism and tissue levels, uncovering a sexual dimorphism where females age earlier but at a slower rate, whereas males age later but experience a faster aging process. To identify terms and themes predictive of age and mortality, unsupervised topic modeling was employed. As a proof of concept, they cross-referenced these terms in PubMed and identified nintedanib as a potential intervention for aging. They demonstrated that nintedanib reduces markers of cellular senescence, mitigates profibrotic gene pathways in senescent cells, and extends the lifespan of fruit flies (unpublished data). These findings open the door to further exploration of population data sets to discover novel interventions for aging.

Martin Vyhnalek and Katerina Veverova, Charles University, Czech Republic, presented their findings from the longitudinal study of aging and dementia, the Czech Brain Aging Study. This complex, clinically based study includes a large data set of measures, including multiple questionnaires, cognitive assessment with a complex neuropsychological battery, CSF and blood-based biomarkers, brain magnetic resonance imaging, amyloid positron emission tomography (PET) imaging, single-photon emission computed tomography (SPECT), *APOE*, and *brain-derived neurotrophic factor* (*BDNF)* genotyping. Patients are followed annually until they convert to dementia. They presented the results of a joint project within the Czech–Norwegian grant in collaboration with the Fang lab in Oslo, focusing on the detection of markers of autophagy/mitophagy and lysosomal degradation in blood and CSF. They found an increase in serum and CSF PINK1 and serum BNIP3L and a decrease in serum TFEB in individuals with AD compared to CU individuals. Mitophagy impairment is correlated with cognitive impairment and amyloid and P-tau biomarkers. The data suggest a potentially nuanced link between common autophagy/mitophagy proteins and AD pathology (collaboration with the Fang lab, data not published).

### Interventions in Aging and Age-Related Diseases

Kristina Xiao Liang, University of Bergen, Norway, described her lab’s use of patient-derived human iPSCs to model neurodegeneration (41,62–64). She explained the versatility of iPSC systems, which include 2D cultures, coculture systems, and 3D culture, such as 3D brain organoids. The core of her talk revolved around the use of iPSC culture as a powerful system for mechanistic and interventional studies, which she has exploited to study the connection between mitochondria and aging, underscoring how aging affects mitochondrial efficiency and contributes to neurodegenerative conditions. Additionally, she highlighted the potential of iPSCs not only in modeling diseases but also in applications such as drug development and tissue regeneration, offering valuable insights into the role of iPSCs in advancing research and treatments for neurodegenerative diseases and beyond.

Linda Hildegard Bergersen, University of Oslo, Norway, discussed the relationship between health, exercise, and neurogenesis, showing in a clinical trial how daily physical activity, which enhances blood flow to the brain and reduces blood pressure (and in some cases also weight) can reduce age-related neuronal loss and decrease the risk of neurodegenerative diseases with age. Exercise also reduced amyloid and phospho-tau load and improved survival and quality of life for patients with AD. Recently, her group showed that plasma from individuals who have exercised can promote neurogenesis in the hippocampus of mouse models of AD (65). She commented on the plethora of clinical trials showing how exercise can improve the lives of subjects with AD. Finally, she highlighted how lactate may be of use for the creation of an “exercise pill.”

He-Ling Wang, University of Oslo, Norway, highlighted the alarming global statistics on AD and the importance of targeting mitochondrial health, discussing NAD^+^ precursors (19) and exercise (65) as potential interventions/treatments. Her research, in collaboration with J.Z. (Central South University and University of Oslo), in *C elegans* models showed that exercise alone (through swimming training), NAD^+^ alone, or a combination of exercise plus NAD^+^, all improved healthspan, mitochondrial function, and memory (66), while the dual regime of exercise and nicotinamide riboside supplementation also protected against aldicarb-induced paralysis; a combination of exercise plus NAD^+^ has additive/synergistic effect compared to each approach (data not published).

Efstathios S. Gonos, Hellenic Pasteur Institute, Greece, presented research on proteasome activation’s role in delaying aging and addressing neurodegenerative diseases. He discussed impaired proteasome function in aging cells and demonstrated methods to activate proteasomes, extending cell lifespan. His work explored proteasome activation’s potential in reducing toxic protein aggregates in diseases like AD. Gonos introduced personalized antiaging strategies and highlighted botanical extracts’ effectiveness in cell culture (67). He also discussed compounds that protect against oxidative stress-induced DNA damage. These discoveries may offer promising avenues for extending healthspan and combating age-related diseases.

Dag Aarsland, King’s College London, UK, discussed various facets of dementia treatment, including current drug therapies, the drug pipeline for AD, and the significance and considerations of new anti-amyloid monoclonal antibodies such as lecanemab and donanemab. He also explored treatment approaches for Lewy body disease, the potential for prevention through lifestyle changes, and the role of the Norwegian Anti-Dementia Drug Trial Platform (NORADD-TP) (68). He highlighted the development of 3 new anti-amyloid drugs, promising early results with anti-tau and anti-alpha synuclein drugs, and the potential of repurposed drugs and supplements. Tormod Fladby, Akershus University Hospital, Norway, shared his experiences in clinical AD diagnosis as well as progress in identifying accurate AD biomarkers. Both D.A. and T.F. stressed the importance of precision in interventions for dementia patients, providing a comprehensive overview of the evolving landscape of dementia treatment and its promising future developments.

## Society and Aging

The public symposium convened at Domus Bibliotheca, University of Oslo, on September 18, 2023, brought together a panel of experts, encompassing scientists, clinicians, and advocates committed to tackling the intricacies of aging and neurodegenerative diseases. Panelists include L.P., V.A.B., M.G.S., D.C.R., N.T., R.S., O.P.O. (University of Oslo and Karolinska Institutet), L.S.C., E.F.F., G.S., J.S.-M. (University of Oslo), alongside individuals who are concerned and eager to address the challenges posed by aging and neurodegenerative diseases.

The central theme of the panel debate was “Anti-Aging Strategies and a Happy Aging Society.” The event, organized into 2 sections, with Section 1 delving into the fundamental aspects of aging research, such as the ethical considerations surrounding the study, popular topics in aging science, and the comparative analysis of animal models and human subjects. Section 2 focused on societal readiness for an aging population, exploring ongoing initiatives, evaluating their efficacy, and discussing the intricate interactions among scientists, politicians, clinicians, and the general populace. The deliberate shift from theoretical considerations to actionable insights underscored the symposium’s commitment to fostering a direct and meaningful connection between the scientific community and the well-being of society, emphasizing the collaborative effort required to improve the lives of individuals facing the complexities of aging and age-related disease. The panel leader Prof. O.P.O. concluded that we are in the right time to perform healthy aging research, an efficient and close collaboration among all the different stakeholders will bring significant and positive socioeconomic impacts.

## Conclusion

The symposium brought together 40 prominent biomedical researchers and clinicians in a hybrid format (with both physical and virtual attendees) to discuss recent breakthroughs in understanding the mechanisms of aging and dementia, as well as insights on clinical treatments and development of novel interventions. The discussions highlighted the promise of new research that may be able to extend healthspan, and targeting age-related diseases by clearing senescent cells, restoring energy homeostasis and DNA repair, preventing pathological protein aggregation, and other novel interventions, as well as personalizing diet and exercise to best promote individual health. The complexity and diversity of human aging mechanisms require innovative and collaborative approaches involving academia, clinical medicine, and industry. This first Norway–UK meeting on aging and dementia has made a start in promoting such collaborations between researchers of the 2 nations and more globally, with the potential for long-term societal benefits from better later life health.
